# ‘PARAMEDIC-2: Big study, small result’

**DOI:** 10.1007/s12471-019-1302-x

**Published:** 2019-07-05

**Authors:** E. ter Avest, H. Lameijer

**Affiliations:** 1 Department of Emergency Medicine, University Medical Center Groningen, Groningen, The Netherlands; 2 Air ambulance trust, Kent, Surrey and Sussex, Redhill airfield, Redhill, Surrey, UK; 3 Department of Emergency Medicine, Medical Center Leeuwarden, Leeuwarden, The Netherlands

**Keywords:** Adrenaline, Out-of-hospital cardiac arrest, OHCA

## Abstract

The PARAMEDIC-2 trial demonstrated that the use of adrenaline compared with placebo in out-of-hospital cardiac arrest (OHCA) resulted in a small increase in 30-day survival, but was associated with a higher number of survivors with severe neurological impairment. These findings received a lot of attention, and generated a widespread discussion about the role of adrenaline in cardiac arrest. In this point of view, we aim to place the PARAMEDIC-2 results in the right perspective by comparing the relative effect of adrenaline to other determinants of cerebral blood flow.

## Point of view

Last year, the authors of the PARAMEDIC2 trial demonstrated that the use of adrenaline compared with placebo in out-of-hospital cardiac arrest (OHCA) resulted in a small increase in 30-day survival. In addition, they showed that no significant between-group difference in the rate of a favourable neurologic outcome was observed, as more survivors had severe neurologic impairment in the adrenaline group [1]. These findings generated a widespread discussion around the use of adrenaline during cardiac arrest, and resulted in the publication of multiple commentaries focusing on the potential beneficial- or detrimental effects of adrenalin in OHCA [2–4].

**Fig. 1:**
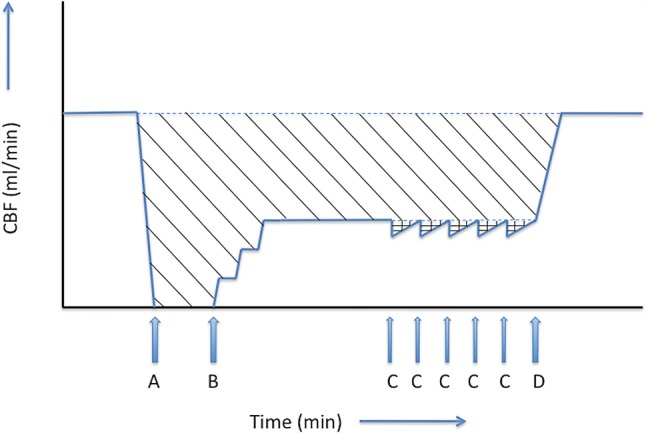
Cerebral blood flow during cardiopulmonary resuscitation. Neurologic outcome of out-of-hospital cardiac arrest (OHCA) is dependent on oxygen delivery to the brain, and thereby on cerebral blood flow (CBF). CBF drops sharply to 0 ml/min during cardiac arrest (**a**). Chest compressions reinitiate CBF, but CBF will not reach pre-arrest levels (**b**). Adrenaline administration during cardiopulmonary resuscitation (**c**) may result in a reduction of the (already compromised) CBF for the duration of the half-life time of the adrenalin. When return of spontaneous circulation (ROSC) is reached, cerebral blood flow is (gradually) restored to normal levels (**d**). The oxygen debit of the brain during the period of arrest is correlated to the total amount of ‘missed blood flow’ during the arrest, which is the product of the difference in cerebral blood flow before- and during the arrest and the total duration of the arrest (*shaded area*). The relative contribution of adrenaline to this area (*crossed area*) is only small

In our opinion, the focus of attention has been too much on the potential detrimental effects of adrenaline on neurological outcome in the aftermath of the trial. Neurological outcome after OHCA is dependent on oxygen delivery to the brain neurons. The cumulative oxygen debit of the brain during the period of arrest is related to the total amount of ‘missed microcirculatory cerebral blood flow’ (CBF) during the arrest, which is the product of the difference in microcirculatory CBF before and during the arrest and the total duration of the arrest (Fig. 1). Although adrenaline has been shown to improve overall CBF, it has a negative effect on cerebral microcirculation [2, 5].

Although this provides a pathophysiological mechanisms for the reported association between adrenaline and a bad neurological outcome in the PARAMEDIC-2 trial, other determinants of microcirculatory CBF likely had a far greater impact on neurological outcome: 37% of the arrests in the PARAMEDIC-2 trial were unwitnessed, and patients received on average 21 minutes of CPR before a first bolus of adrenaline was administered. These prolonged no-flow and resuscitation times likely had a far greater impact on neurological outcome than the (average dose of 4.9 mg) adrenaline administered (Fig. 1). This is supported by the (albeit not reported) high number needed to harm for adrenaline in the PARAMEDIC-2 trial: 39/4015 patients survived with modified Rankin score of 4 or 5 in the adrenaline group compared with 16/3999 in the placebo group, resulting in a number needed to harm of 175.

In our opinion, attention should therefore be focused on the improvement of bystander CPR and early defibrillation rather than focusing on marginal gains (or pains) of adrenaline administration during OHCA.

## References

[1] Perkins GD, Ji C, Deakin CD (2018). A randomized trial of epinephrine in out-of-hospital cardiac arrest. N Eng J Med.

[2] Kuiper MA (2018). Epinephrine, a double edged sword?. Ned. Tijdschr. Geneeskd.

[3] Jung J, Rice J, Bord S (2018). Rethinking the role of epinephrine in cardiac arrest: the Paramedic2 trial. Ann Transl Med.

[4] Cook R, Davidson P, Martin R (2019). Adrenaline can restart the heart, but is no good for the brain. BMJ.

[5] Gough CJR, Nolan JP (2018). The role of adrenaline in cardiopulmonary resuscitation. Crit Care.

